# Advanced measurement and diagnosis of the effect on the underlayer roughness for industrial standard metrology

**DOI:** 10.1038/s41598-018-36991-z

**Published:** 2019-01-31

**Authors:** Jung-Hwan Kim, Seunghyun Moon, Ji-Woong Kim, Donggun Lee, Byong Chon Park, Dal-Hyun Kim, Yoojin Jeong, Sean Hand, Jason Osborne, Peter De Wolf, Youn Sang Kim, ChaeHo Shin

**Affiliations:** 1 Scientific Instruments Reliability Assessment Center/Smart Open Lab., Korea Basic Science Institute, Daejeon, 34113 Republic of Korea; 2 Division of Industrial Metrology, Korea Research Institute of Standards and Science, Daejeon, 34113 Republic of Korea; 3 Program in Nano Science and Technology, Graduate School of Convergence Science and Technology, Seoul National University, Seoul, 08826 Republic of Korea; 4 Bruker Semiconductor, 112 Robin Hill Road, CA Santa Barbara, 93117 USA; 5 Advanced Institutes of Convergence Technology, 864-1 Iui-dong Yeongtong-Gu, Suwon-si, Gyeonggi-do, 16229 Republic of Korea; 6 Advanced Instrumentation Institute, Korea Research Institute of Standards and Science, Daejeon, 34113 Republic of Korea

## Abstract

In current nanoscale semiconductor fabrications, high dielectric materials and ultrathin multilayers have been selected to improve the performance of the devices. Thus, interface effects between films and the quantification of surface information are becoming key issues for determining the performance of the semiconductor devices. In this paper, we developed an easy, accurate, and nondestructive diagnosis to investigate the interface effect of hafnium oxide ultrathin films. A roughness scaling method that artificially modified silicon surfaces with a maximum peak-to-valley roughness range of a few nanometers was introduced to examine the effect on the underlayer roughness. The critical overlayer roughness was be defined by the transition of RMS roughness which was 0.18 nm for the 3 nm thick hafnium oxide film. Subsequently, for the inline diagnostic application of semiconductor fabrication, the roughness of a mass produced hafnium film was investigated. Finally, we confirmed that the result was below the threshold set by our critical roughness. The RMS roughness of the mass produced hafnium oxide film was 0.11 nm at a 500 nm field of view. Therefore, we expect that the quantified and standardized critical roughness managements will contribute to improvement of the production yield.

## Introduction

In relation to current industrial semiconductor metrology, the management of the thickness of ultrathin films has been conducted in a strict manner. Electron microscope studies, such as vertical scanning electron microscopy (VSEM) and transmission electron microscopy (TEM), are crucial to accurately measuring the thickness of ultrathin films and calibrating optical thickness measurement tools. These instruments are used to measure the films directly, but practical uses are difficult for inline metrology due to the potential damage to the device during the destructive sampling process^1–4^.

Ellipsometry is a common approach to managing the thickness of the transparent and translucent films used in the semiconductor manufacturing process because this method is fast and nondestructive^5,6^. The film thickness can be calculated from measuring optical constants, such as the reflection coefficients and phase changes, by detecting the polarized light reflected from the thin film. Although spectral ellipsometry is a powerful tool when managing film thicknesses at the angstrom (Å) level using a proper micrometer spot size on the illuminated areas, it does not provide sub-nanoscale local surface information due to the limited lateral resolution associated with this method^5,7^.

Atomic force microscopy (AFM) is an indispensable semiconductor metrology tool that is capable of reliably and accurately observing surface structures nondestructively^8–13^. It directly assesses the surface morphology and roughness with a sub-nanometer spatial resolution. For example, the metal etch-back (MEB) depth profiles of dynamic random-access memory (DRAM), for which no signal can be detected by spectroscopic ellipsometry (SE), can be examined by using inline automated AFM (AAFM). Other examples include: scanning of the channel holes of flash memory devices and the surfaces of solder or copper pillar bump materials, measurements of the critical dimensions of the mask after development inspection (ADI) processes, and inspections to determine electrical failures in self-aligned contact (SAC) or landing pad (LP) processes in DRAM modules. In addition, roughness examinations of the ultrathin films without any interference from the lower membrane have been implemented^12,14^.

In recent years, a great deal of attraction has been paid to improve the performance of the AFM because of the surge of interest in the ultrathin films. Many efforts have been made to replace the gate SiO_2_ (K = 3.9) layer with a high dielectric constant (K) to reduce the tunneling current and ensure low power consumption for a complementary metal-oxide semiconductor (CMOS)^15–20^. Among high dielectric constant materials, hafnium oxide (HfO_2_) films are being used in the semiconductor industry due to their relatively high K values (K = 25) and large band gaps^21^. However, the reliability of HfO_2_ thin films comes into question on multiple layers due to the poor interfacial quality and inferior thermal stability between the Hf atoms^22–25^. Therefore, providing the criteria pertaining to sub-nanoscale surface roughness has become an important task.

We report herein an easy, accurate, and nondestructive diagnosis of the effect on the underlayer roughness for industrial standard metrology regarding surface roughness. The surface roughness levels of HfO_2_ thin films were analyzed by a low-noise (LN) AFM system. The surfaces of the substrates were artificially modified using a wet etching process to examine how the underlayer roughness affects the overlayer roughness, and the relationship between each fabrication step was investigated. The critical roughness (CR) criterion of the HfO_2_ thin film can be established according to the intersection between two linear fits. After that, for the inline diagnosis of semiconductor fabrication, the roughness of a mass produced hafnium film was investigated. We assessed the roughness of a mass-produced HfO_2_ wafer by using in-line AAFM and LN AFM. From the results, we confirmed that the roughness of a mass-produced HfO_2_ wafer with a thickness of 3 nm is below our CR value. The average value of the root mean square (RMS) roughness is 0.11 nm with a 500 nm field of view (FOV), and the dynamic repeatability and reproducibility (R&R) value is 30 pm (the 3 σ value for 10 measurements) for the in-line inspections. Such a quantified and standardized management of critical roughness by AFM metrology for a few nanometers thin films will help to improve production yield and establish industrial standard metrology.

### Optimal environmental conditions for roughness measurements using a low-noise (LN) AFM system

We constructed a LN AFM system (RMS noise ~35 pm) optimized for surface metrology in the semiconductor industry and the uncertainty was estimated by a quantitative method (see Supplementary Table 1 in Supplementary Information)^26–28^. The reliability of surface information is now an essential factor because the results of these measurements can be easily distorted depending on the environment and probe conditions. Low vibration of the tip is necessary for high-resolution AFM imaging, which is ensured by keeping the distance between the tip and the sample as small as possible. In this experiment, the relative humidity (RH) was held below 10% by injecting highly pure argon gas, as the measured force can be interrupted by water layers on the substrate^29–31^. The distance between the tip and the sample could be held constant at 4.4 nm.

**Figure 1:**
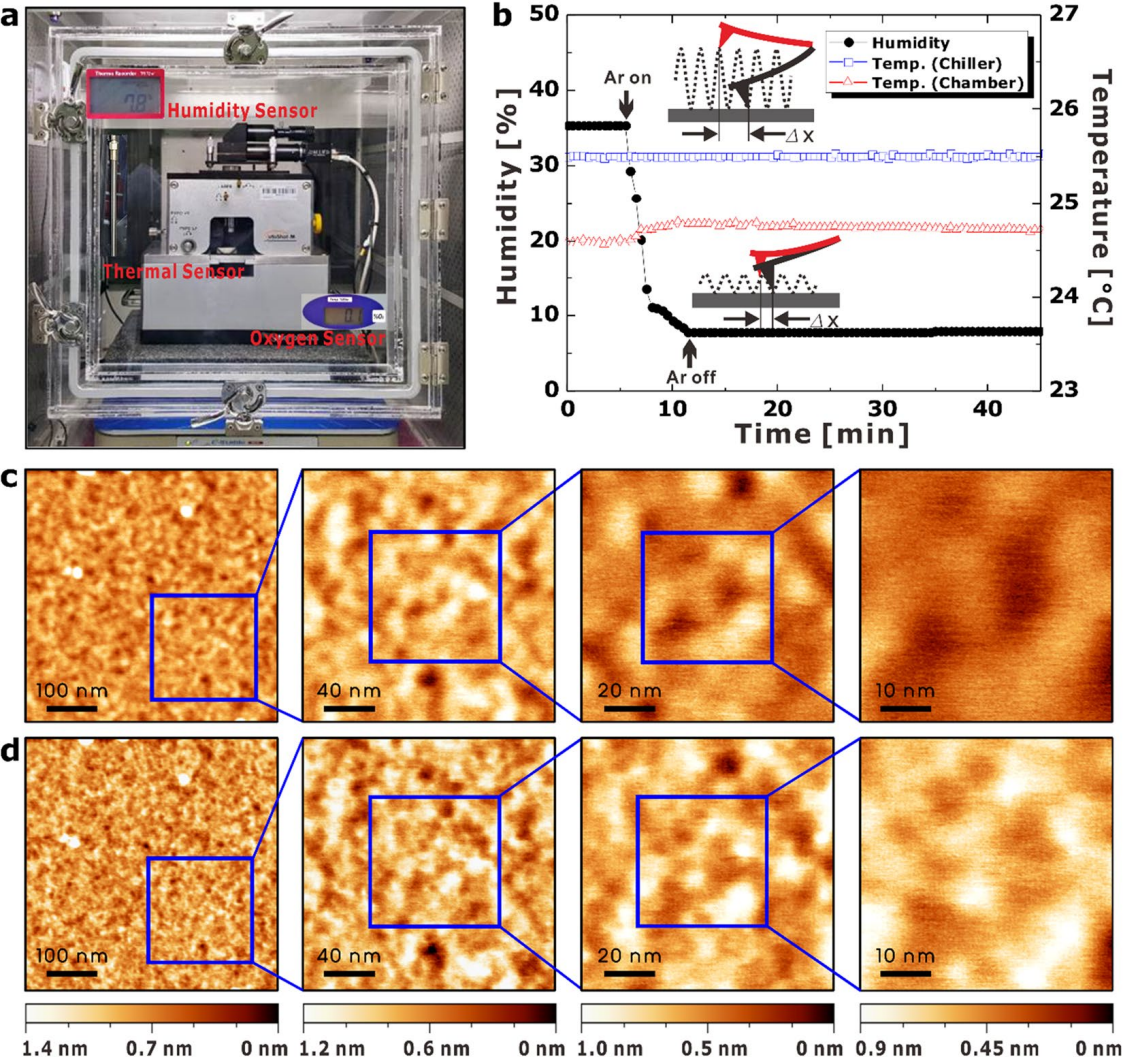
(**a**) LN AFM system equipped with humidity, thermal, and oxygen sensors. The oxygen sensor is used for safety purpose. (**b**) Plot of the humidity and temperatures in the AFM measurement environments. (**c**) AFM images of HfO_2_ film at a relative humidity of 35%. The RMS roughness is 0.13 nm at a 500 nm FOV. (**d**) AFM images of HfO_2_ film at a low humidity level of less than 10%. The RMS roughness is 0.15 nm at a 500 nm FOV at a position identical to that in panel (c).

Figure 1 presents the effects of an argon injection on the humidity and shows the differences in the lateral resolution with different field-of-view (FOV) values. At RH 35%, relatively blurry images were obtained, as shown in Fig. 1c, because the motion of the cantilever is strongly affected by water molecule layers on the sample surface. Conversely, under dry conditions, the apex of the cantilever can come very close to the sample surface such that clearer images can be taken, as presented in Fig. 1d. For this reason, the RMS roughness in the dry environment is higher than that in the relatively humid atmosphere. The lateral resolution of the images is high enough to distinguish nanostructures, the grain size of which is less than 10 nm, as shown in Fig. 1d. Ultimately, the LN AFM system was able to visualize the roughness of the HfO_2_ film at a nanoscale FOV.

### Study of interfacial effects using the roughness scaling method

**Figure 2:**
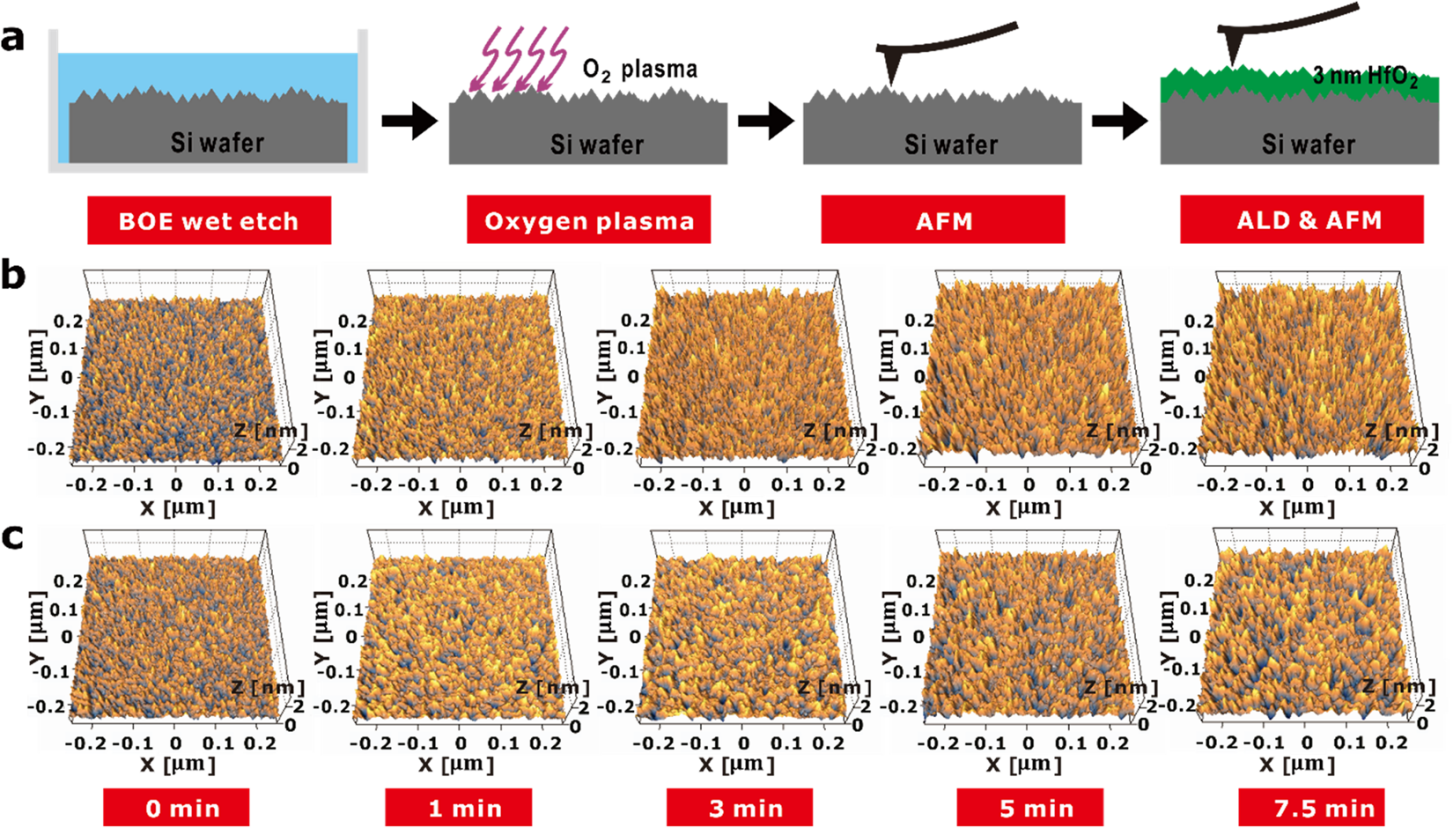
(**a**) Schematic illustration of the roughness scaling method. Roughness scaling (Si substrates) was carried out using a BOE solution with different dipping times. (**b**) Representative AFM images of roughened silicon surfaces taken after oxygen plasma treatments. (**c**) AFM images of hafnium oxide surfaces after atomic layer deposition (3 nm in thickness).

A wet etching process was used to provide a variety of roughness levels of silicon (Si) substrates. The surface roughness was regulated by changing the dipping time in a buffered oxide etchant (BOE) solution (see Supplementary Fig. 1). In order to examine the spatial resolution by using LN AFM in a range of a few nanometers, the roughness of the Si surface was increased until the maximum peak-to-valley value (*R*_*t*_) approached 3 nm. This process is designated as the “roughness scaling method” in Fig. 2. The samples that did not undergo the BOE process are denoted as “0 min” in Fig. 2b,c. An oxygen plasma treatment was conducted after surface etching because the hydrogen-terminated Si surface shows poor nucleation and forms a nano-island morphology during the atomic layer deposition (ALD) process^23,32–35^. An HfO_2_ layer was deposited onto the surface-treated Si wafers using ALD equipment (Nano-ALD2000; IPS, Pyeongtaek, Korea) because the insulating film of the mass-produced wafer is a 3 nm thick hafnium oxide film, as shown in Supplementary Fig. 2. Tetrakis (ethylmethylamino) hafnium (TEMAHf) and ozone (O_3_) were used to form the hafnium oxide layer at 350 °C. The gas pressure was 0.7 Torr. The surfaces of each of the samples were measured with the LN AFM instrument before and after the hafnium oxide deposition process.

**Figure 3:**
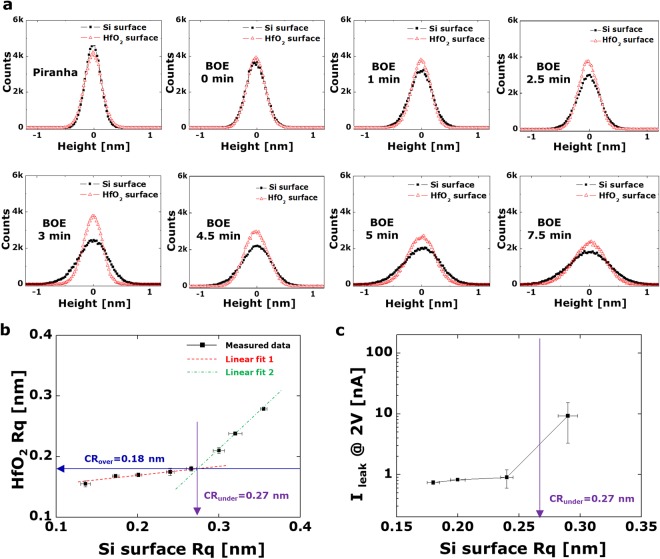
(**a**) Histograms of the height distributions of each AFM image at 500 nm FOV. (**b**) RMS roughness of a silicon surface before the ALD process vs. a hafnium oxide surface for five measurements. The first linear fit data (slope 0.13, intercept 0.14 nm) was extracted from the four lowest data values. The second fit data (slope 1.24, intercept −0.16 nm) was extracted from three highest data values. (**c**) Current at 2 V from the MIM diode structure with different RMS roughness of the Si substrate.

In order to determine the difference between the initial and final states of the surface roughness, the height distributions are overlapped in the plots, as illustrated in Fig. 3a. The parameters extracted from these distributions, in this case the arithmetic average of the roughness (*R*_*a*_), the RMS roughness (*R*_*q*_), and the distance between the highest peak and the lowest valley (*R*_*t*_), are listed in Table 1. The RMS roughness and the *R*_*t*_ values are also plotted in Fig. 3b and Supplementary Fig. 3, respectively. As shown in Fig. 3b and Table 1, the result can be distinguishable into two groups. On the relatively smooth substrates, there is no significant effect on the roughness of the HfO_2_ overlayer. It can be explained as the smoothing phenomenon during ALD process that was also observed in other studies^25,36^. However, the RMS roughness of the HfO_2_ overlayer is dramatically increased when the *R*_*t*_ values of the rough silicon surface approaches the thickness of the hafnium oxide film. This phenomenon is significantly related to the conformal growth of the ALD process since the surface variations are sufficiently high^37,38^. In other words, the final roughness is less affected by the underlayer (Si substrates) unless the *R*_*t*_ values of the Si substrates match the thickness of the HfO_2_ film. Thus, we can determine the critical roughness based on the experimental data in Fig. 3b. Two linear fits were conducted to find each slope and intercept. The parameters of the first linear fit data were calculated by selecting the five lowest data values. The parameters of the second fit data were extracted from the three highest data values, as shown in Fig. 3b. The critical roughness can be determined from the intersection between the two linear fits, as described by the following equations:

1 $${{{\rm{CR}}}_{{\rm{over}}}|}_{t=3\,nm}^{Hf{O}_{2}}=\frac{{b}_{1}-\alpha {b}_{2}}{1-\alpha },\,(\alpha \equiv \frac{{a}_{1}}{{a}_{2}})$$

2 $${{\rm{CR}}}_{{\rm{under}}}=\frac{1}{\beta }({b}_{1}-{b}_{2}),\,(\beta \equiv {a}_{2}-{a}_{1})$$

where *t* is the thickness of the hafnium oxide film, *a*_1_ and *b*_1_ are correspondingly the slope and the intercept extracted from the first linear fitting function, and *a*_2_ and *b*_2_ are likewise the slope and the intercept of the second linear fitting function. The finally estimated CR value of the HfO_2_ overlayer in this dielectric system was 0.18 nm while the CR value of the underlayer was 0.27 nm. Moreover, in order to verify the effectiveness of the CR, we measured the leakage current through the metal-insulator-metal (MIM) diode structure^39,40^. As shown in Fig. 3c, the leakage current was rapidly increased after the CR. (see Supplementary Fig. 4 for current density plot of minimum, median, and maximum data). Therefore, the CR value defined by the simple method in this study proved to be effective. Although the CR value in this experiment is not an absolute criterion for all hafnium oxide films, we believe that the proposed method will be useful for establishing an industry standard CR.

**Table 1:** Roughness information (shown in Fig. 3a) for surface control samples at a 500 nm FOV.

	Surface	*R*_*a*_ [nm]	*R*_*q*_ [nm]	*R*_*t*_ [nm]
Piranha	Silicon oxide	0.11	0.14	1.15
	Hafnium oxide	0.12	0.16	1.34
BOE 0 min	Silicon oxide	0.14	0.17	1.47
	Hafnium oxide	0.13	0.17	1.35
BOE 1 min	Silicon oxide	0.16	0.20	1.93
	Hafnium oxide	0.14	0.17	1.36
BOE 3 min	Silicon oxide	0.21	0.27	2.45
	Hafnium oxide	0.14	0.17	1.49
BOE 5 min	Silicon oxide	0.25	0.32	2.98
	Hafnium oxide	0.19	0.24	2.07
BOE 7.5 min	Silicon oxide	0.28	0.36	3.24
	Hafnium oxide	0.22	0.28	2.19

### Inline morphology analysis of ultrathin hafnium oxide films for industrial metrology

During the semiconductor fabrication process, surface morphology measurements of the sub-nanoscale roughness are among the most challenging applications in a typical facility environment. They require a high performance of the anti-vibration efforts and acoustic shielding while executing atomic force feedback control on the surface. In addition, there are many items to be considered to obtain reliable data^41,42^. Thus, we undertook a surface analysis of a mass-produced ultrathin HfO_2_ film sample (~3 nm in thickness) using an optimized LN AFM system. Subsequently, roughness measurements of an identical mass-produced HfO_2_ wafer were conducted using an inline AAFM system (InSight; Bruker Corporation, USA), including an auto-level stage, automated probe exchange, and thermal stability, the process of which is fully automated for the mass production of these wafers. A highly accurate laser interferometer stage was used for the measurements. The resonance frequency of the AAFM itself and the fingerprint frequency of the environmental noise in the semiconductor facility were separated to achieve sub-nanoscale roughness with the in-line AAFM system. The background noise level was approximately 35 pm.

The roughness parameters calculated from each topography image are summarized in Table 2. All roughness parameters are similar except for that in the area with a low FOV of 50 nm. A small scanning area (50 nm FOV) led to differences due to the lateral resolution limit in that case. The average value of the RMS roughness is 0.11 nm and the dynamic repeatability and reproducibility (R&R) value is below 30 pm (the 3σ value for 10 measurements). There are minor differences between the two results, as shown in Table 2. This subtle difference is considered to be caused by the different measurement environments and by the tip conditions. Thus, the results showed good agreement with the LN AFM outcome within the tool performance specifications.

**Table 2:** Roughness parameters extracted from each topography image (shown in Fig. 4b).

**Figure 4:**
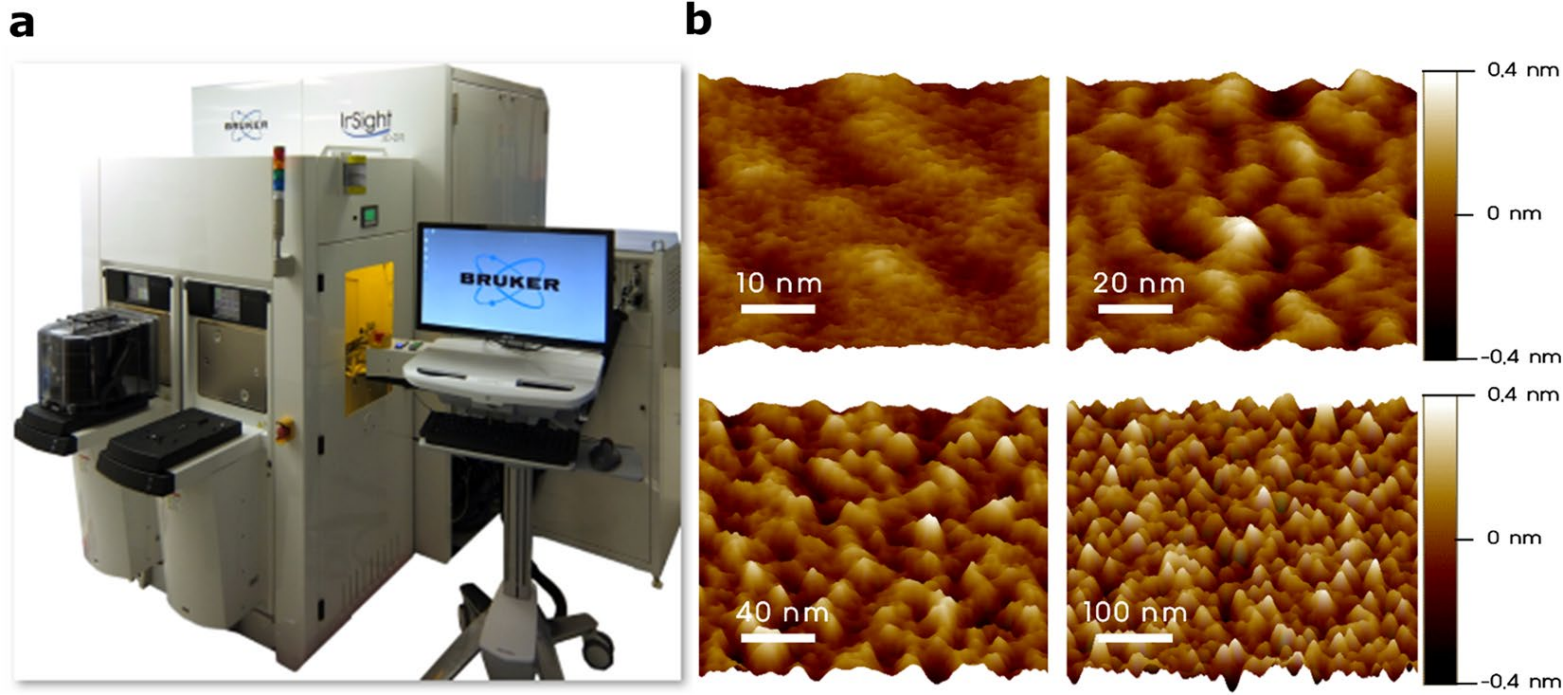
(**a**) Inline production auto-AFM. (**b**) AFM image of a hafnium oxide thin layer demonstrating the capability of roughness measurements in a fabrication facility (Fab) environment.

	FOV [nm]	*R*_*a*_ [nm]	*R*_*q*_ [nm]	*R*_*t*_ [nm]
LN AFM	50	0.09	0.11	0.80
	100	0.10	0.13	0.82
	200	0.09	0.12	0.84
	500	0.09	0.12	0.98
Inline AAFM*	50	0.06	0.08	0.72
	100	0.09	0.11	0.75
	200	0.09	0.11	0.78
	500	0.08	0.10	0.83

*Note: image isolation was performed for inline AAFM data.

## Discussions

In the current semiconductor manufacturing process, the thickness parameter of ultrathin films is strictly managed on a test element group (TEG) between the chips by using spectroscopic ellipsometry. However, the thickness metrology of ultrathin films on a TEG does not reflect the actual characteristics of cell patterns; thus, on-cell metrology is required to provide more reliable surface information^43^. As the thickness of the dielectric layers becomes thinner, the task of providing the criteria pertaining to the critical roughness has attracted considerable attention, particularly in relation to surface roughness variations on devices designed with dimensions of a few nanometers. An example of this is the side wall roughness for three-dimensional nanostructures^44^, such as a fin field-effect transistor (FinFET) device. Moreover, the metrology on the side wall roughness and the effect of the underlayer on the surface wall after depositing a very thin layer remain challenging issues.

The present study utilized roughness scaling metrology for an ultrathin dielectric layer, hafnium oxide film, as utilized in the semiconductor manufacturing process. The effect of the substrate roughness on the roughness of a 3-nm-thick hafnium oxide overlayer was investigated using low-noise AFM. The overlayer roughness (HfO_2_ films) is less affected by the low roughness of Si substrates, but the interface effect became severe only when the maximum peak-to-valley parameter of the initial substrate was close to the thickness of the hafnium film (underlayer R_t_ ~ 3 nm). Thus, the CR value can be determined from the intersection of the two linear fits. The CR values of the overlayer and the underlayer were 0.18 nm and 0.27 nm, respectively. In addition, the effectiveness of the defined CR was confirmed by measuring the leakage current of the MIM structure.

We also confirmed the effects of sub-nanometer roughness management in an actual fabrication environment by applying an inline AAFM technique developed for a mass-produced monitor. The dynamic repeatability and reproducibility (R&R) of the roughness information for mass-produced HfO_2_ films are in good agreement with those of LN AFM. Hence, the results here will contribute to development, enhance the yields of next-generation semiconductor devices, and ensure reliable standardization metrology for surface roughness levels.

## Methods

### Low-noise (LN) AFM measurements

The custom-built LN AFM system used here was developed at KRISS (the Korea Research Institute of Standards and Science). The temperature (22.3 ± 0.1 °C) of the LN AFM system can be controlled by circulating temperature-controlled liquid. An ultrathin HfO_2_ surface was inspected in tapping mode using a high-density carbon probe (SuperSharpStandard-NCHR; Nanotools, Germany) with a normal probe radius of ~3 nm and a cantilever spring constant of 40 N/m. The tip diameter of the manufacturer specifications is below 10.0 nm. The cantilever was oscillated at 5.28 nm (free-air amplitude) with a Q-value of 537. The set point of the distance between the probe and the sample was 4.4 nm. When the AFM probe was engaged on the sample, we kept the set point at 7 nm for a wide separation distance to reduce or prevent damage to the probe. The probe was then slowly moved toward the sample in 0.1 nm steps with a large proportional and integral gain factor.

### Piranha sample preparation

Si wafers were immersed in a piranha solution (a 3:1 mixture of H_2_SO_4_ and H_2_O_2_) for 30 min and rinsed with deionized water. Oxygen plasma was implemented at a pressure level of 100 mTorr for 90 sec. Subsequently, a hafnium oxide film sample with a thickness of 3 nm was deposited onto the Si surface.

### Roughness control with BOE wet etching

Si substrates underwent a sonication process in acetone and isopropyl alcohol for 30 min each. The substrates were then gently rinsed with deionized water. The wet etching process was performed at room temperature using a 30:1 buffered oxide etchant (BOE) solution. The etched substrates were then cleaned again with deionized water. The oxygen plasma treatments were carried out at a pressure of 100 mTorr for 90 sec. Subsequently, the HfO_2_ films were deposited onto the roughened surface.

### Inline AAFM measurements

An ultrathin HfO_2_ surface was inspected in tapping mode using a silicon probe (RTESPA-300; Bruker, USA) with a normal probe radius of 8 nm and a cantilever spring constant of 40 N/m. The cantilever was oscillated at 25 nm (free-air amplitude) and the set point was 15 nm. In order to be used in semiconductor fabrication, the probe is very quickly engaged on the sample with a digital signal processor control within a few seconds to ensure no damage to the probe. The temperature variation in the acoustic shielding chamber was held under 0.1 °C at room temperature. Vibration was eliminated through feedback from the special linear motor used with a granite air-floating and anti-vibration table. The AFM feedback motion was conducted by changing the amplitude of the oscillating probe with an image isolation™ function. This method isolates the background frequency and the special fingerprint frequency from the environment before the image scan.

## Supplementary information

Supplementary Information
