# Supplementary material for: Advanced measurement and diagnosis of the effect on the underlayer roughness for industrial standard metrology
Source: Sci Rep. 2019 Jan 31;9:1018. doi: 10.1038/s41598-018-36991-z (PMC6355768; doi:10.1038/s41598-018-36991-z)
Supplement: Supplementary file 1 — Supplementary Information [file 41598_2018_36991_MOESM1_ESM.docx]

**Supplementary Information**

**Advanced measurement and diagnosis of the effect on the underlayer roughness for industrial standard metrology**

**Jung-Hwan Kim^1^**^†^**, Seunghyun Moon^2,3^**^†^**, Ji-Woong Kim^4^, Donggun Lee^3^, Byong Chon Park^2^, Dal-Hyun Kim^2^, Yoojin Jeong^4^, Sean Hand^4^, Jason Osborne^4^, Peter De Wolf^4^, Youn Sang Kim^3,5*^, and ChaeHo Shin^2,6*^**

1 Instrumentation Development Support team, Korea Basic Science Institute, Daejeon, 34113, Republic of Korea

2 Division of Industrial Metrology, Korea Research Institute of Standards and Science, Daejeon, 34113, Republic of Korea

3 Program in Nano Science and Technology, Graduate School of Convergence Science and Technology, Seoul National University, Seoul, 08826, Republic of Korea

4 Bruker Semiconductor, 112 Robin Hill Road, CA, Santa Barbara, 93117, USA

5 Advanced Institutes of Convergence Technology, 864-1 Iui-dong Yeongtong-Gu, Suwon-si, Gyeonggi-do, 16229, Republic of Korea

6 Advanced Instrumentation Institute, Korea Research Institute of Standards and Science, Daejeon, 34113, Republic of Korea

^†^ These authors contributed equally to this work.

* Prof. Youn Sang Kim (E-mail: [younskim@](mailto:younskim@)snu.ac.kr)

* Dr. ChaeHo Shin (E-mail: [chaeho.shin@kriss.re.kr](mailto:chaeho.shin@kriss.re.kr))

**1. Uncertainty for roughness measurements**

The candidates of the noise sources of the LN AFM are the equipment itself and the environment. The noise of the facility can be managed with a very constant level, and the measurements using the LN AFM were tried to prevent the external environmental influences. Thus, we measured five different sites (upper, lower, left, right, and center position) of the mass produced hafnium oxide film to estimate the uncertainty for the roughness measurements. In addition, we obtained the reliable measurement values from z-piezo value using a tilt stage of LN-AFM (not shown here for the tilt stage). According to the Guide to the Expression of Uncertainty in Measurement (GUM), the combined uncertainty ($R_{2}$) can be expressed by below relation.

$$u_{c}\left( R_{2} \right) =\sqrt{R_{1}^{2}*u^{2}\left( K \right)+\bar{K^{2}}\left\{ {u^{2} (R}_{a})+u^{2}(R_{b})+{u^{2}(R}_{c})+{u^{2}(R}_{d}) \right\}} ,$$

where *u*(*K*) is the calibration coefficient, $R_{a}$ is the uncertainty of caused by the repeatability measurement, $R_{b}$ is the uncertainty of caused by the non-uniformity (5 different sites) of specimen, $R_{c}$ is the uncertainty of instrument noise of z-feedback without x-y motion, $R_{d}$ is the uncertainty of tip wearing during measurement, *R*_1_ is the average value of RMS roughness measurement, and $\bar{K}$ is the average calibration coefficient of the instrument.

**Supplementary Table 1. Summary of uncertainty**

| **Source of uncertainty** | **Contribution of each uncertainty source** |
| --- | --- |
| Calibration coefficient, u(*K*) | 0.012 |
| Repeatability, u($R_{a}$) | 0.003 nm |
| Non-uniformity, u($R_{b}$) | 0.003 nm |
| Instrument noise, u($R_{c}$) | 0.035 nm |
| Tip wearing, u($R_{d}$) | 0.003 nm |
| u($R_{2}$) | 0.036 nm |

**2. Roughness scaling**


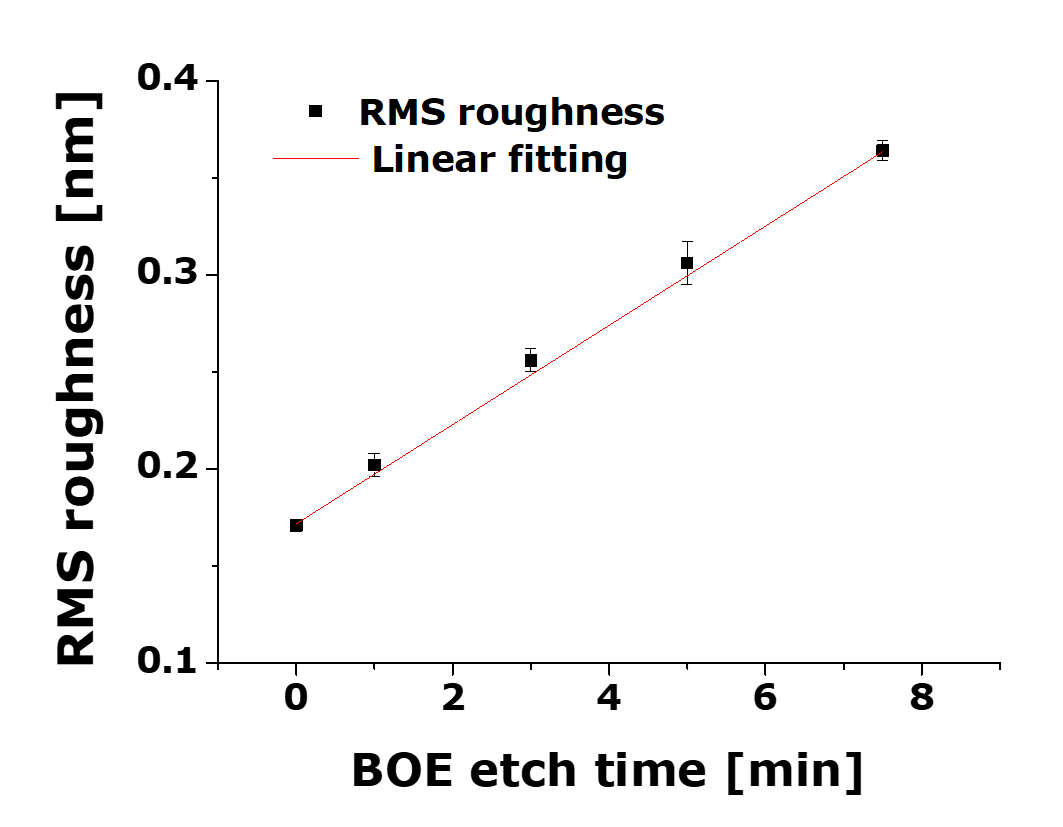


Supplementary Figure 1. This plot shows how to determine the underlayer roughness range in the roughness scaling method.

When determining the scaling range, the *R_q_* value of the sample subjected to the O_2_ plasma process on the bare Si substrate was designated as the minimum point. The R_q_ value of the bare Si substrate was 0.15 nm and increased to 0.17 nm after the O_2_ plasma treatment. The maximum roughness was set to *R_q_* = 0.36 nm because the maximum peak-to-valley values reached the film thickness when dipped in the BOE solution for more than 7.5 minutes.


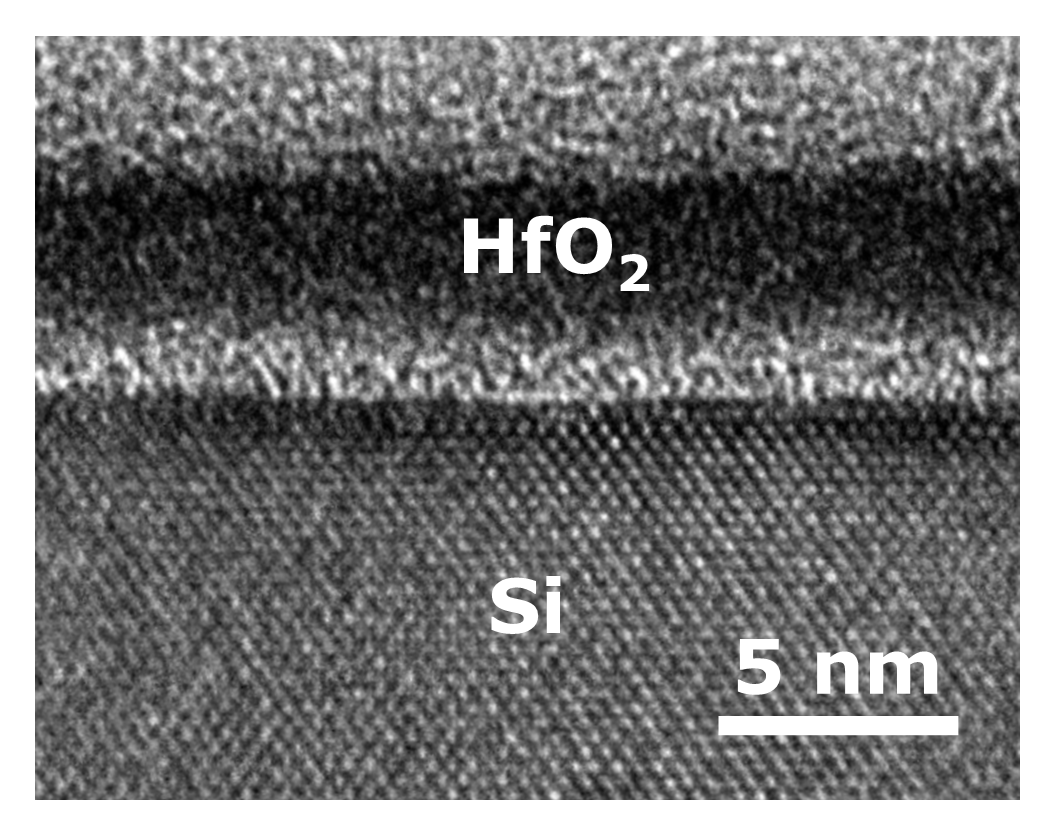


Supplementary Figure 2. TEM image of mass-produced hafnium oxide film with 3 nm thickness.

The high-resolution transmission electron microscopy (TEM) image of the hafnium oxide film was taken with a JEM-2100F (JEOL, Tokyo, Japan). The thickness of the HfO_2_ film was calculated to be approximately 3 nm by DigitalMicrograph (Gatan, Pleasanton, CA, United States) software.


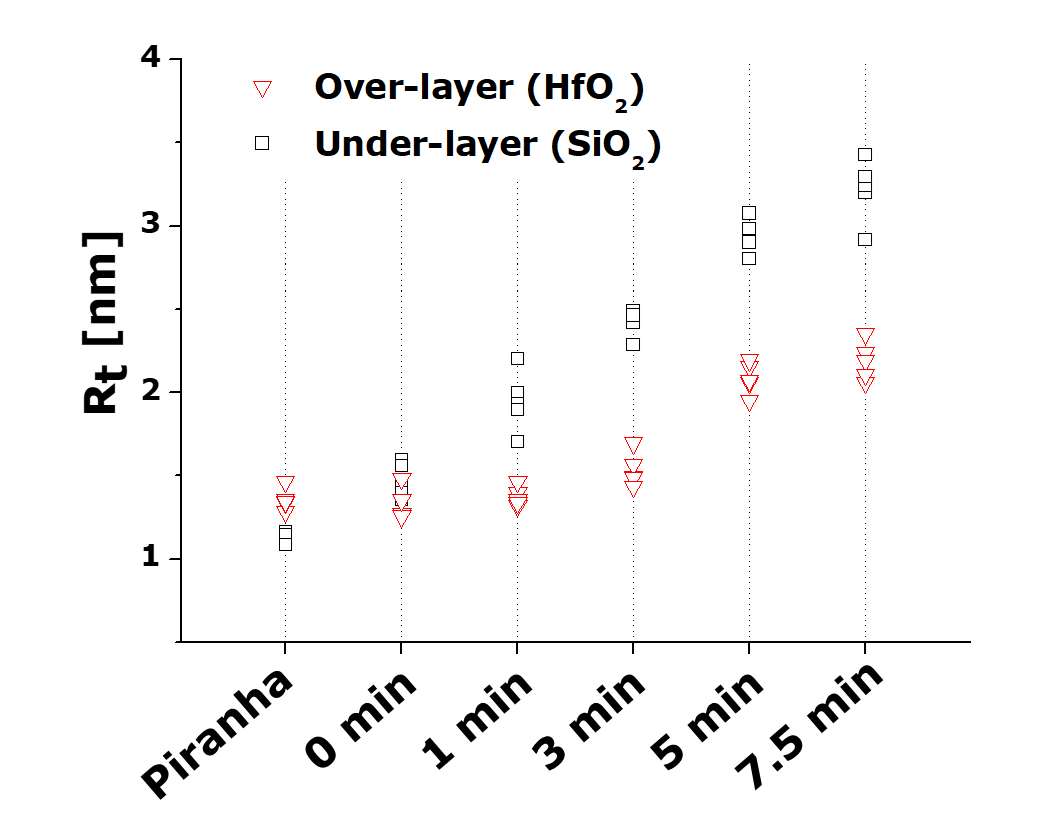


Supplementary Figure 3. Maximum peak-to-valley (R_t_) distributions of AFM images for 5 measurements. Each measurement was taken before and after the ALD process. The black square data represent the R_t_ values of silicon oxide surface. The red triangle data show the R_t_ values of hafnium oxide films.

As shown in Supplementary Fig. 3, the piranha-treated sample was named “Piranha” instead of BOE etch process. The other samples were immersed in BOE solution at different times. The AFM images of the underlayers (SiO_2_) were measured 5 times for each sample after the oxygen plasma. The AFM images of the overlayers (HfO_2_) for each sample were measured 5 times at different points after the ALD process.

**3. Leakage current measurements using metal-insulator-metal diode structure**


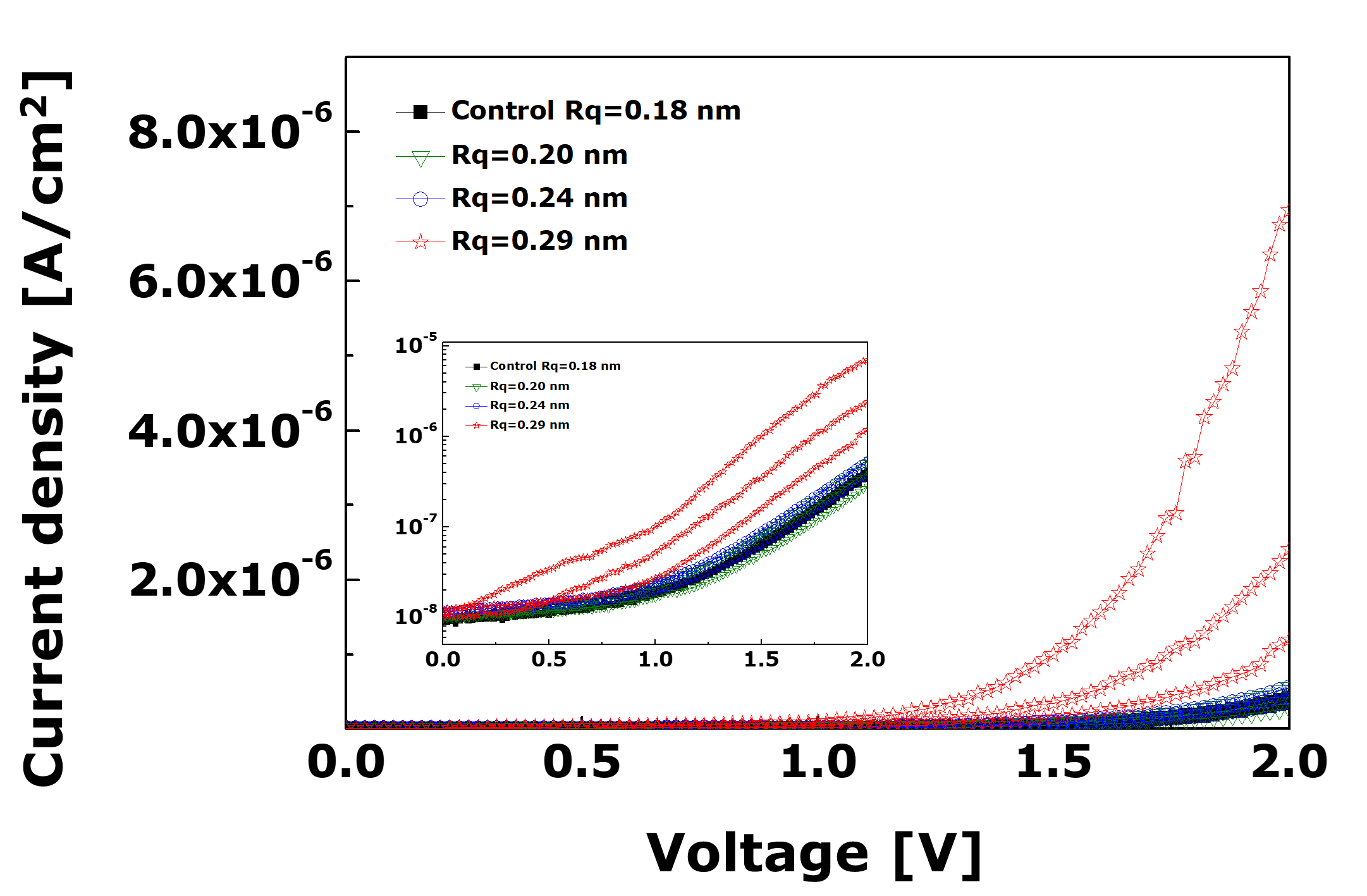


**Supplementary Figure 4.** Current density plot of the minimum, median, and maximum data. (inset) A logarithm plot of the current density.

The MIM diodes were fabricated to measure the leakage currents. A highly doped Si wafer was used for the substrates of the MIM diodes. In the case of highly doped Si wafers, it took a long time to adjust the roughness by the BOE wet etching, so the roughness was controlled by the KOH wet method. The control sample was subjected to O_2_ plasma treatment without etching, and the hafnium oxide film (3-nm-thick) was fabricated by ALD method. Subsequently, the aluminum film (50-nm-thick) was deposited by a thermal evaporator to complete the MIM structure. The diameter of the aluminum pad metal is 500 μm. A two-probe system was used for the current measurement and the average value was evaluated through 20 different devices. SFig. 4 is the IV plot of minimum, median, and maximum values ​​among 20 devices, and is drawn with different Si roughness. As shown in the logarithm plot, almost all devices show constant current before the CR, whereas the current of the device with a larger roughness than the CR is increased.
